# *Paederus* dermatitis

**DOI:** 10.1007/s15010-024-02231-y

**Published:** 2024-03-13

**Authors:** Laura C. Kalkman, Omrimaxwell M. Sesay, Martin P. Grobusch

**Affiliations:** 1 Center of Tropical Medicine and Travel Medicine, Department of Infectious Diseases, Amsterdam University Medical Centers, Location Amsterdam University, Meibergdreef 9, 1205 AZ Amsterdam, The Netherlands; 2 Masanga Medical Research Unit, Masanga, Sierra Leone

**Fig. 1:**
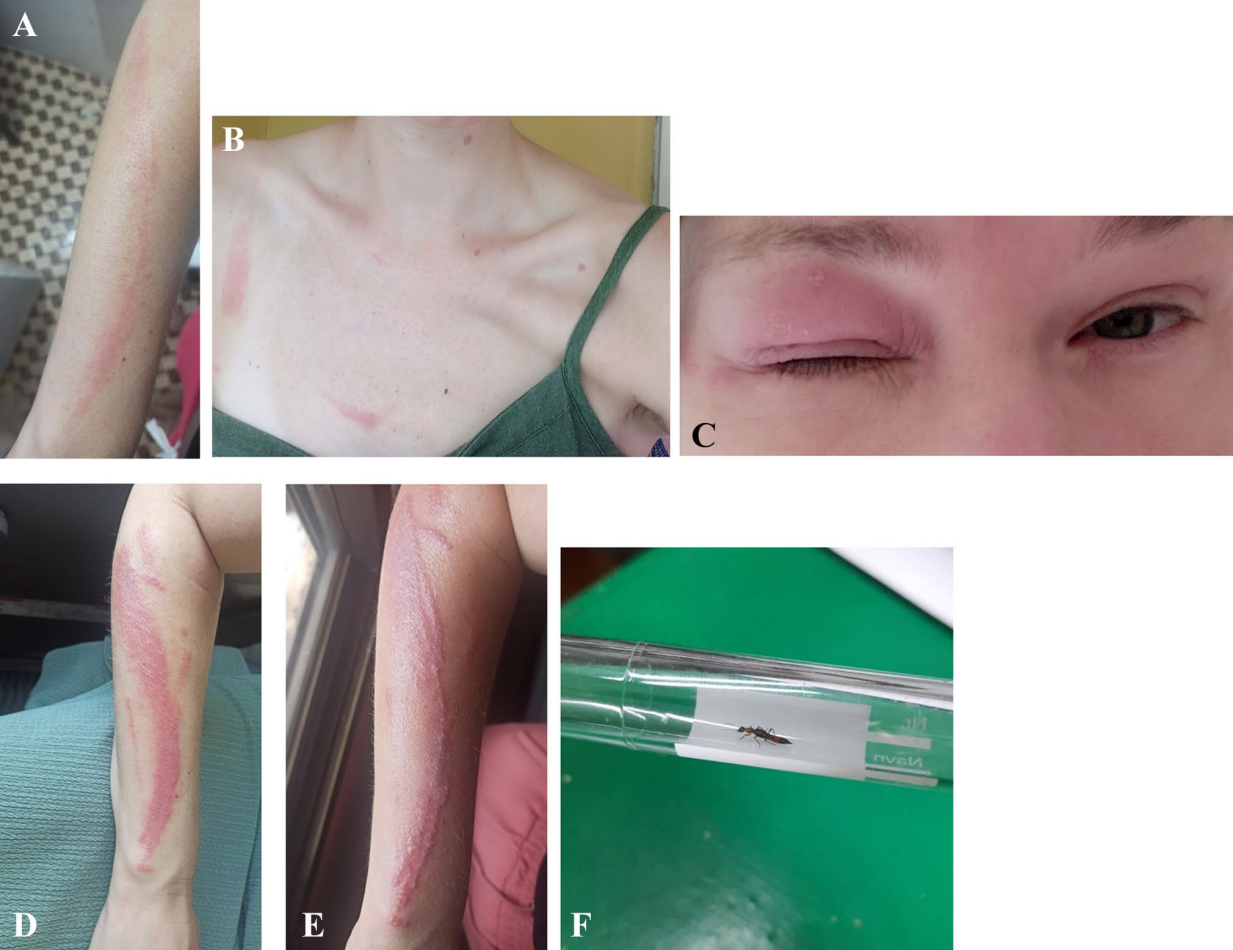
Dermatitis linearis. **A** Secondary erythematous lesions Day 1. **B** Faint erythematous lesion Day 1. **C** Erythema and swelling of eyelid. Day 1. Dermatitis on Day 4. **E** Dermatitis on Day 5. **F** *Paederus* beetle collected on site in Masanga, Sierra Leone

A 31-year-old woman of European origin living in rural Sierra Leone developed a faint erythematous lesion on the right forearm at the beginning of the rainy season. Simultaneously, two similar lesions appeared on the chest along the clothing line and on the right eyelid, causing eyelid swelling without any symptoms of conjunctivitis (Fig. 1A–C). Over the course of four days, the lesions on the chest and eyelid subsided, while the lesion on the forearm progressed to a painful erythematous dermatitis (Fig. 1D, E), resembling a lesion caused by exposure to a toxic substance. The skin lesions presented as linear erythematous plaques, with vesicles and bullae appearing at the periphery of the lesion. No systemic symptoms occurred. Pain and inflammation subsided after applying a class III topical corticosteroid.

The diagnosis, ‘champion fly bite’, was made by a local health worker. This is the local term for a condition also referred to as ‘*Paederus* dermatitis’, blister beetle dermatitis’, or, amongst other terms, ‘dermatitis linearis’. It is caused by the rove beetle, i.e. many different *Paederus* species across a wide geographical range across the Middle East, Africa, Asia, the Americas, and Australia [1]. It must be noted that the term ‘dermatitis linearis’, however, is considered ambiguous by some, as it might be used as well for Lichen striatus, an inflammatory skin disease with often similar ‘linear’ appearance. In fact, with erythematous plaques, and the presence of vesicles, subcorneal pustules and bullae [1], the dermatological differential diagnosis of this toxic dermatitis should be considered as broad, including other forms of contact dermatitis, phytophotodermatitis, thermal and chemical burns, impetigo bullosa; even herpes zoster and herpes simplex might have to be considered depending on case-individual distribution pattern and appearance of the lesions [2].

*Paederus* beetles are most active in humid and warm weather conditions, and outbreaks have been described in East Africa (‘Nairobi fly’), West Africa, South America, North America, Australia and the Middle East. *Paederus* beetles are slender, reach a length of 7–10 mm, with a width of 1.5–2 mm, are black in colour, with an orange-red thorax and lower abdominal segment [1, 2] (Fig. 1F).

The causative agent of the skin laesions is the very potent pederin toxin and its analogues, stored in the haemolymph of the beetle—not produced by the beetle itself but by endosymbiontic *Pseudomonas* spp. [1–3]. The toxin is transferred when it is crushed on the skin (designations as ‘bite’ or ‘sting’ are misnomers in colloquial language). At the time of contact, the beetle itself remains often unnoticed. Lesions most often occur in the face, elbow, or armpit, where the beetle is crushed. The toxin can be transferred to other regions of the body if the individual touches these areas after crushing the beetle, such as in this case the chest and the eyelid. Treatment includes washing the affected area after exposure; and applying topical corticosteroids. The most frequent endpoint of the healing process is *restitutio ad integrum*; however, scarring is possible and does not occur infrequently [1, 2]. *Paederus* dermatitis can be prevented by brushing insects off and screening doors and windows, as they are attracted to light [4].
